# Prediction of hip joint function and analysis of risk factors for internal fixation failure after Femoral Neck System (FNS)

**DOI:** 10.1186/s12891-023-06805-z

**Published:** 2023-08-24

**Authors:** Yazhong Zhang, Xu Zhang, Chao Li, Yan Lin, Yongxiang Lv, Shaolong Huang, Bin Wang, Yunqing Wang, Ziqiang Zhu

**Affiliations:** Department of Orthopaedics, The Second Affiliated Hospital of XuZhou Medical University, No 32 Meijian Road, Xuzhou, 221000 Jiangsu China

**Keywords:** Femoral Neck System, Femoral neck fractures, Hip joint function, Internal fixation failure, Shortened femoral neck

## Abstract

**Objective:**

Analysis of the risk factors affecting hip function and complications after femoral neck system (FNS) surgery for femoral neck fractures is of great significance for improving the procedure’s efficacy.

**Methods:**

The data of patients with femoral neck fractures who underwent FNS surgery in our hospital between October 2019 and October 2020 were retrospectively analyzed. Age, gender, time from injury to operation, fracture classification, operation time, fracture reduction, and postoperative weight-bearing time information were set as potential factors that may affect the results. Hip Harris scores were performed at 12 months postoperatively, and postoperative complication data (e.g., femoral head necrosis, nonunion, and femoral neck shortness) were collected. The risk factors affecting hip function and complications after FNS surgery were predicted using linear and logistic regression analyses.

**Results:**

A total of 69 cases of femoral neck fracture were included, with an average age of 56.09 ± 11.50 years. The linear analysis demonstrated that the age and fracture type of the patients were the risk factors affecting the Harris score of the hip joint after FNS surgery. Older patients with displaced femoral neck fractures had an inferior postoperative hip function. In addition, fracture type, reduction of the femoral neck, and postoperative weight-bearing significantly impacted postoperative complications. Displaced fractures, negative fixation, and premature weight-bearing (< 6 weeks) were risk factors for postoperative complications. The Harris score of patients with a shortened femoral neck in the included cases was not significantly different from that of patients without shortening (*P* = 0.25).

**Conclusions:**

Advanced age and fracture type are important evaluation indicators of the Harris score after FNS internal fixation of femoral neck fractures in young patients. Fracture type, fracture reduction, and postoperative weight-bearing time are risk factors for complications after FNS.

## Introduction

Internal fixation surgery remains the preferred option for young patients with femoral neck fractures [1, 2]. As a new fixation method for treating femoral neck fractures, FNS has been gradually popularized and applied due to its minimal invasiveness and stability. In our previous short-term follow-up, we compared the clinical efficacy of FNS and parallel cannulated screws in young patients with femoral neck fractures and revealed that FNS offers a considerable advantage [3].

Due to the special anatomy of femoral neck fractures, the fracture end faces irregular rotational and shear forces. In addition, the fragile blood supply of the femoral neck is also an important factor in the higher failure rate after internal fixation [4]. The recovery of hip function affects the quality of life of patients and survival rate to a large extent. Many studies on multiple cancellous or sliding hip screws have proved that factors such as patient age, fracture type, posteromedial support, reduction quality, posterior tilt [5], operation time, BMD (Bone Mineral Density), and other factors may affect the healing of femoral neck fractures [6]. However, no studies have analyzed the risk factors of FNS in treating femoral neck fractures.

Therefore, since the recovery of hip joint function and the occurrence of complications after operation are affected by many aspects, predicting the risks related to the efficacy of FNS has extraordinary guiding significance for improving the success rate of the surgery.

## Methods

### Data collection

Data on patients with femoral neck fractures who underwent FNS surgery in our hospital between October 2019 and October 2020 were collected. Our retrospective study included patients with at least 12 months of follow-up data. FNS was obtained from DePuy Synthes (Zuchwil, Switzerland). Data collection and follow-up assessments were performed by two experienced trauma surgeons. The data collected included age, gender, fracture type, time from injury to operation, operation time, fracture reduction, postoperative weight-bearing time, and complications. Postoperative complications include femoral head necrosis, nonunion, and neck shortening. The surgical procedure refers to our previous study [3]. In this study, all patients underwent closed reduction using a surgical table. The position of FNS bolt is located in the three-dimensional center of the femoral neck, which is operated according to FNS standard surgical procedures. This study was approved by the institutional review board ([2019]080701), and all patients signed an informed consent form.

**Fig. 1:**
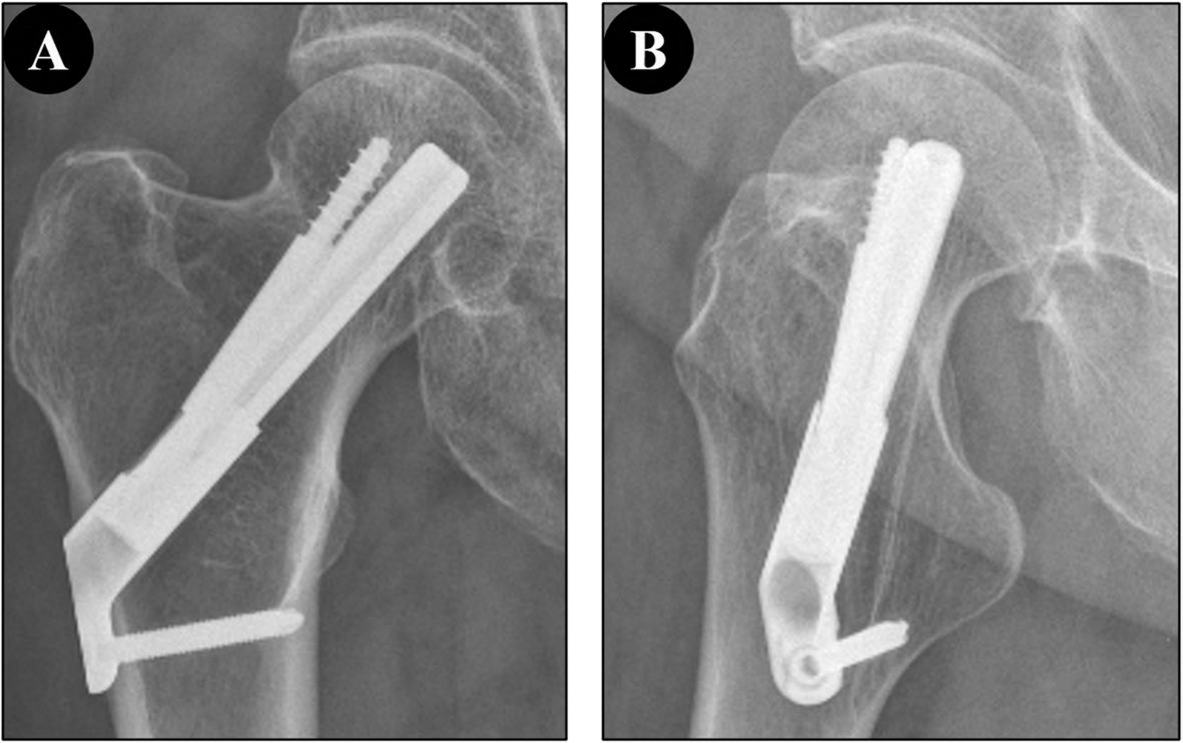
**A** and **B** Hip joint frontal and axial X-rays treated with FNS

FNS surgical images were shown in Fig. 1.

### Criteria for inclusion and exclusion

Inclusion criteria were patients diagnosed with a fresh closed femoral neck fracture who underwent FNS surgery and completed at least 12 months of postoperative follow-up. Exclusion criteria were hip dysfunction before the injury, fractures in other parts, or other diseases affecting the treatment of the femoral neck (e.g., pathological fractures or rheumatoid diseases), loss of follow-up, or incomplete data.

### Fracture type

The fracture types of patients were evaluated by preoperative X-ray and computed tomography (CT). In this study, all femoral neck fractures were divided into displaced and undisplaced types according to the degree of displacement and stability of the fracture. According to the commonly used Pauwels and Garden classification systems, the displaced types mainly include PauwelsII, PauwelsIII, GardenIII, and GardenIV, and the undisplaced types include PauwelsI, GardenI, and GardenII types [7, 8].

### Reduction of femoral neck fractures

Through postoperative X-ray fluoroscopy, the reduction was determined according to the position of the fracture end. The reduction was divided into anatomical reduction, positive fixation (the distal inner edge of the fracture protrudes inwardly and downwardly compared with the proximal inner edge of the fracture), and negative fixation (the distal inner edge of the fracture protrudes outwardly and upwardly compared with the proximal inner edge of the fracture).

### Postoperative weight-bearing

There was no uniform quantitative index for postoperative weight-bearing. In our study, patients were defined as premature post-operative weight-bearing within six weeks of vertical contact of the operated leg with the ground from the first postoperative day. Our definition is that the formation of new bone at the fracture ends can already be observed by CT at six weeks. Early weight bearing destroys the newly formed callus. Sitting and standing on the unaffected leg alone were permitted.

### Follow up and hip research indices

Harris scores of the two patient groups were evaluated at 12 months after surgery. The Harris score (HHS) system assesses four aspects: pain, function, absence of deformity and range of motion. The score standard had a maximum of 100 points. Total scores < 70 points were considered poor scores, 70–80 points were fair, 80–90 points were good while 90–100 points were excellent scores.

### Statistical analysis

The SPSS version 23.0 (SPSS Inc. Chicago, IL, USA) was used for statistical analyses. Hip function scores and complications occurrence were considered dependent variables, while observed measures were used as independent variables. Data are reported as the mean ± standard deviation (SD), and categorical variables are expressed as percentages. Linear analysis was performed to identify risk factors affecting hip function scores and analyze the correlation between postoperative hip joint function and age in displaced group and undisplaced group; unconditional logistic regression was performed to identify risk factors for a complication, 95% confidence intervals were calculated, and the null hypothesis Odds Ratio (OR) = 1 was checked using the Wald test. Differences were considered statistically significant at *p* < 0.05.

## Result

### Demographic data

A total of 69 patients participated in the study and completed at least 12 months of follow-up and data collection. The average age of the 69 patients was 56.09 ± 11.50 years, including 46 males and 23 females. Other observed data included time from injury to operation, fracture classification, operation time, fracture reduction, postoperative weight-bearing time (< 6 weeks or not), and the Harris score. Detailed data are presented in Table 1. The surgical procedure refers to our previous study.

**Table 1:** Demographic data

Demographic data	
**Age (years, mean ± SD)**	56.09 ± 11.50,
**Gender (n, %)**	Male:46(66.67, %), Female:23(33.33%)
**Fracture type (n, %)**	Displaced:27(39.13%), Undisplaced:42(60.87%)
**Time from injury to surgery (hour, mean ± SD)**	27.14 ± 9.39
**Surgical time (min, mean ± SD)**	53.17 ± 14.83
**Reduction of femoral neck fractures (n, %)**	Anatomical reduction:45(76.27%), Positive buttress:14(20.29%), Negative buttress:10 (14.49%)
**Postoperative weight-bearing (n, %)**	<6 weeks:34 (49.28%), >6 weeks:35 (50.72%)
**Harris score (mean ± SD)**	80.74 ± 8.15

### Complications after FNS surgery

Of the 69 patients, 18 developed complications related to fracture union after FNS fixation, and one patient developed nonunion and necrosis of the femoral head. Table 2 demonstrates the incidence of complications in the study population.

**Table 2:** Complications after FNS

Postoperative complications	(n, %)
**Femoral head necrosis**	5 (7.24%)
**Nonunion**	5 (7.24%)
**Neck shortening**	9 (13.04%)

The effect of risk factors on complications was analyzed using binary logistic regression. Complications (*n* = 19) associated with FNS fixation observed in this study included femoral head necrosis, nonunion, and shortening of the femoral neck. The results are displayed in Table 3. Fracture type (*P* < 0.01), reduction situation (*P* = 0.04), and postoperative weight-bearing (*P* = 0.01) have significant effects on complications (*P* < 0.05).

**Table 3:** Influence of the factors on the complications

Factors	P-value	Exp(B)	95% confidence interval
**Age**	0.24	1.041	0.97 to 1.11
**Gender**	0.29	0.42	0.87 to 2.05
**Fracture type**	< 0.01	25.25	3.62 to 176.10
**Time from injury to surgery**	0.22	1.06	0.97 to 1.16
**Surgical time**	0.59	1.02	0.97 to 1.07
**Reduction of femoral neck fractures**	0.04	0.10	0.01 to 0.89
**Postoperative weight-bearing**	0.01	17.78	2.10 to 150.75

### Hip functional results

**Fig. 2:**
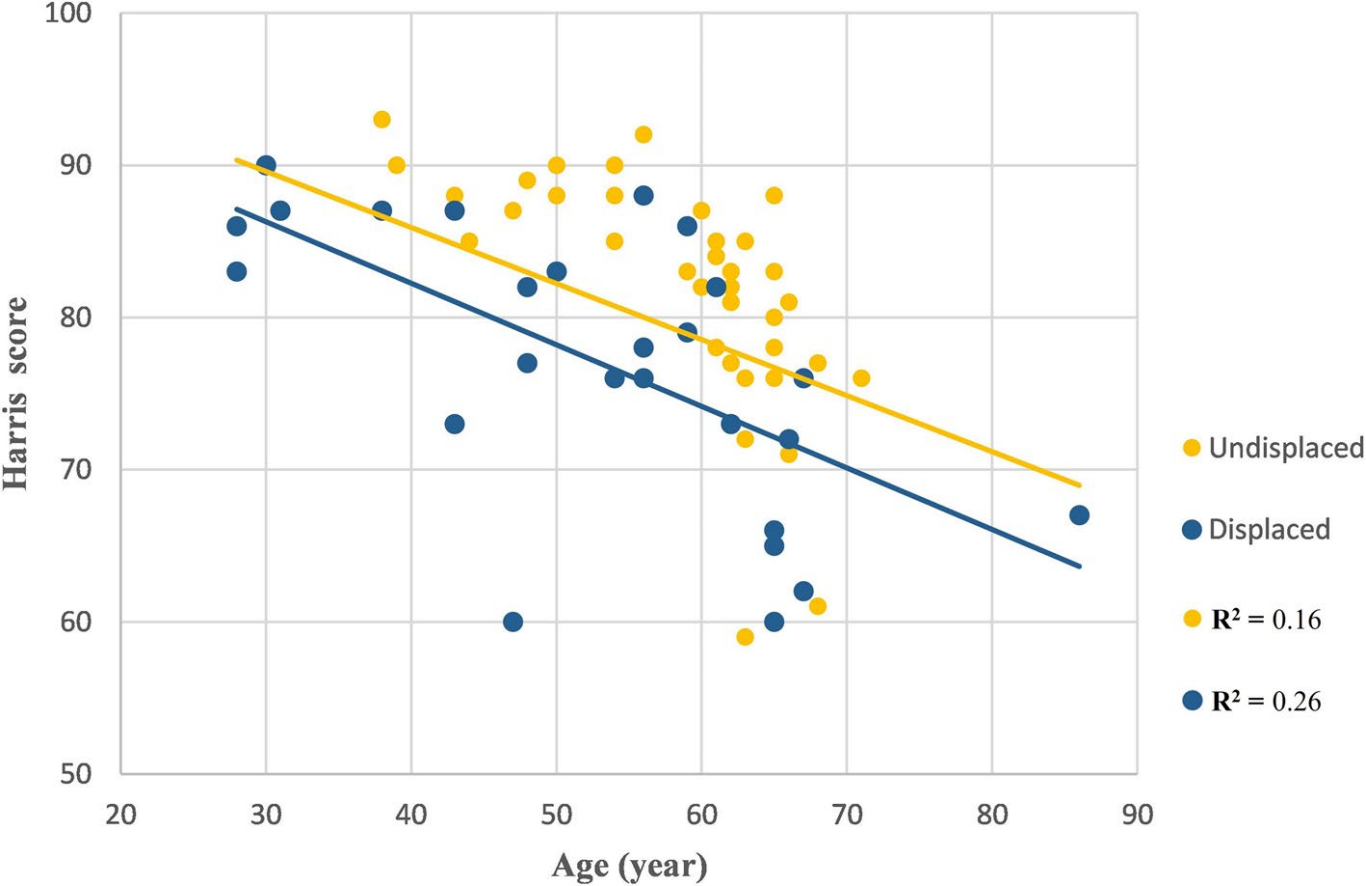
Graphical representation of the effect of age on the Harris score

Among the potentially influential risk factors, age (*P* < 0.01) and fracture type (displaced and undisplaced, *P* < 0.01) have a significant effect (*P* < 0.05) (Table 4) on the Harris score. Figure 2 shows a graphical representation of the effect of age on the Harris score, where *R*^2^ (unshifted) = 0.16 and *R*^2^ (shifted) = 0.26. As illustrated in Fig. 2, the Harris score gradually decreases with the increase in age, as shown by the linear analysis.

**Table 4:** Influence of the factors on the Harris score

Factors	P-value	95% confidence interval
**Age**	< 0.01	-0.58 to -0.29
**Gender**	0.45	-4.64 to 2.08
**Fracture type**	< 0.01	-11.00 to -3.92
**Time from injury to surgery**	0.51	-0.34 to -0.02
**Surgical time**	0.73	-0.09 to 0.12
**Reduction of femoral neck fractures**	0.48	-3.04 to -6.40
**Postoperative weight-bearing**	0.05	-6.20 to -0.01

We divided the 69 patients into a displacement and non-displacement group according to the fracture type, including 27 and 42 cases, respectively. The difference in the Harris score between the two groups is illustrated in Table 5.

**Table 5:** Influence of fracture type on Harris score

	(n, %)	Harris score (mean ± SD)	t	P
**Displaced**	27(39.13%)	77.33 ± 9.21	2.20	0.03
**Undisplaced**	42(60.87%)	81.71 ± 7.30		

The Harris score is 77.33 ± 9.21 in the displaced group and 81.71 ± 7.30 in the non-displaced group. The postoperative Harris score of patients with undisplaced femoral neck fracture was significantly higher than that of the displaced group (*P* = 0.03).

In our follow-up study, the incidence of femoral neck shortening after FNS is 13.04%. To investigate whether femoral neck shortening after FNS has an impact on the quality of life and hip function, we excluded patients with femoral head necrosis and nonunion and divided the remaining 60 patients according to whether femoral neck shortening occurred or not (*n* = 9 and *n* = 51 for the shortening and unshortened group, respectively). Differences in the Harris scores between the two groups are demonstrated in Table 6.

**Table 6:** Influence of shortening on Harris score

	(n, %)	Harris score (mean ± SD)	t	P
**Shortening**	9(15.00%)	80.55 ± 4.90	-1.17	0.25
**Non-Shortening**	51(58.00%)	82.82 ± 5.42		

## Discussion

The high failure rate of femoral neck fracture procedures in younger patients remains challenging [9]. FNS has been used as a new surgical modality for treating femoral neck fractures. It has several advantages, such as anti-rotation, angular stabilization, and dynamic fixation, and is a minimally invasive operation [10]. However, there is no report on the risk factors affecting hip function and complications after FNS.

We conclude that age and fracture type (displacement or not) were risk factors for the Harris score after FNS, determined using linear analysis. Most studies on the internal fixation of femoral neck fractures depict that increasing age is strongly associated with failure and poorer function [11]. BMD may decrease with age, especially in postmenopausal women, which will affect the fracture repair process [12]. Likewise, our study showed that Harris scores decreased after FNS with increasing age, regardless of the presence of displacement or non-displacement. Due to the special anatomical position and stress characteristics of the femoral neck, different types of fractures have always been a research hotspot. In our study, 69 cases were divided into displaced types (PauwelsII, PauwelsIII, GardenIII, and GardenIV) and non-displaced types. Using linear and logistic regression analyses, the Harris score of displaced femoral neck fractures (77.33 ± 9.21) was significantly lower than that of undisplaced fractures (81.71 ± 7.30) (*P* < 0.05); the incidence of postoperative complications was higher than that of the nondisplaced type. For displaced femoral neck fractures, on the one hand, traction and reduction are required during the operation, which may increase the damage to the soft tissue of the affected limb. On the other hand, prolonged postoperative bed immobilization will not only increase osteoporosis. Further, the incidence of this disease will also lead to some muscle atrophy and the degeneration of knee and ankle joint functions.

Complications associated with FNS fixation observed in this study (*n* = 19) included femoral head necrosis, nonunion, and shortening of the femoral neck. The results revealed that fracture type, reduction, and postoperative weight-bearing were the risk factors for FNS postoperative complications. Displaced fractures are often accompanied by the destruction of the anatomical structure of the fracture end, as the bone at the fracture end is more easily absorbed, and the defect of the medial cortical bone cannot provide effective bone support [13]. Finite element analysis of FNS demonstrated that for the reduction of femoral neck fractures, negative bracing was more prone to the shortening of the femoral neck. Due to the presence of the femoral calcar, the medial and posterior aspect of the femoral neck is thus the key to bone strength. When the femoral neck is in an anatomical position or positive fixation, the pressure of body weight can be effectively transmitted and dispersed downward through the medial arch of the femoral neck, with a certain compression effect. Further, the negative support will face greater shear force due to the lack of effective pressure transmission, which may be the reason for the higher failure rate of the negative support [14, 15].

Due to the difference in rehabilitation guidance provided by the doctors and the medical compliance of the patient, postoperative weight-bearing exercise is a difficult indicator to quantify. In our study, patients who performed weight-bearing activities upright (standing and walking) within six weeks after surgery were defined as premature weight-bearing, with or without a walker. The six-week time node is determined because the X-ray examination can already observe the appearance of a new callus at six weeks as well as during our follow-up process, where the symptoms of pain and swelling of the outer thigh soft tissue have disappeared after the operation. In our follow-up, premature activity within six weeks after surgery consisted mainly of indoor short-distance walking (mainly in the bathroom). The patients with premature weight-bearing in the follow-up population mainly consisted of younger, preoperative physical laborers. During treatment, the surgeon will instruct patients to stay in bed for at least six weeks before discharge and guide them on a weight-bearing exercise program to be carried out after that, according to the results of the first postoperative follow-up X-ray.

Regardless of the type and reduction of the fracture, there is some shear force at the fracture end of the femoral neck. We believe that the shear force of the femoral neck after internal fixation is the key to the healing of the femoral neck. Although many studies have shown that the compression and fretting of the fracture end can promote healing, for the femoral neck, this theoretical healing is under the premise that the shear force is effectively controlled [16]. This is because of the correlation between the medical compliance and disease cognition of the patient, living habits, and even work habits [17]. For displaced femoral neck fractures and patients with long reduction time and unsatisfactory reduction (negative fixation), the risk of internal fixation failure is relatively high, and the surgeon should conduct strict follow-up within six weeks after surgery. Supervision of the patient is needed to avoid premature weight-bearing.

Studies have shown that fracture type, posteromedial cortical comminution, and reduction quality are the main risk factors for neck shortening [18]. In our study, femoral neck shortening, as one of the complications related to internal fixation after FNS, had an incidence of 13.04% (9/69), which was significantly lower than that with screw fixation. Compared with parallel screw fixation, FNS fixation has the advantage of angular stability (i.e., locking and bolt, bolt, and anti-rotation nail). Displaced fractures often have comminuted fractures or cortical bone defects, which increase postoperative bone resorption and thus increase the incidence of postoperative complications, especially the shortening of the femoral neck [19]. All nine patients with femoral neck shortening in this study had shortenings of less than 5 mm, with no significant difference in postoperative hip function scores compared with those without shortening (*P* > 0.05). This also shows that the angular support of FNS can provide good axial support and effectively avoid excessive compression of the femoral neck [10].

It is worth noting the limitations of this study. First, the clinical component of this study was a retrospective analysis with a limited number of cases, which may introduce selection bias; second, due to the limited number of cases, fracture types and postoperative weight-bearing time were not classified in more detail. These issues require further studies with an expanded number of cases and increased long-term, detailed follow-ups.

## Conclusion

Our findings support that age and fracture type are possible predictors of hip function after FNS. Fracture type, reduction, and postoperative weight-bearing are risk factors for postoperative complications following FNS.

## Data Availability

The datasets used and analyzed during the current study are available from the corresponding author on reasonable request.
